# To Derogate or to Restrict? The COVID-19 Pandemic, Proportionality and the Justificatory Gap in European Human Rights Law

**DOI:** 10.1007/s42439-022-00065-6

**Published:** 2022-07-12

**Authors:** Alain Zysset

**Affiliations:** 1 School of Law, University of Glasgow, Glasgow, Scotland; 2 PluriCourts, University of Oslo, Oslo, Norway

**Keywords:** COVID-19 pandemic, Proportionality, Justificatory gap

## Abstract

In this paper, I offer an analytical and normative framework to re-visit the question of whether state parties should derogate from the European Convention on Human Rights (ECHR) in order to combat the COVID-19 pandemic via harsh ‘lockdown’ measures. It is three-pronged. First, I show that the predominant debate on the (non-)derogation question is informed by a textual approach to adjudication, which severely limits the analytical and evaluative horizon for addressing the issue. Most importantly, it cannot address one salient fact about the effects of lockdown measures, namely their highly disproportionate effects on vulnerable groups and minorities. Second, I argue that proportionality assessment should be the basis for determining whether state parties ought to derogate or not. This is because proportionality’s very purpose is in part to track the effects of state interferences on minorities and vulnerable groups by measuring the disproportionate burden imposed on them. It is also because proportionality assessment has very different requirements between limitation clauses built into the relevant Convention articles (e.g. Article 5, Articles 8–11) and the derogation clause (Article 15) under the ECHR. Surprisingly, while the emerging literature almost always mentions proportionality as an important component of the analysis, it does not investigate the extent to which each regime (derogation or limitation) better performs it, and why. Third, I draw from the philosophical literature on the ‘right to justification’ to clarify the egalitarian and justificatory function of proportionality. Unlike derogation, limitation clauses have a much higher and systematic requirement of justification, which makes the case for non-derogation clearer and stronger.

## Introduction

Should state parties derogate from the European Convention on Human Rights (via Article 15) to more effectively combat the pandemic? As a matter of facts, 10 state parties to the ECHR have notified the Council of Europe and have effectively derogated.^1^ This split has further highlighted the normative question of whether derogation is appropriate and/or desirable for public authorities to combat the pandemic of COVID-19. In the UK, a debate has emerged between those who think that the pandemic constitutes an ‘ideal situation’ for derogating (Greene 2021) and those who think that the required interferences with rights can remain within the text of the Convention via a limitation or restriction of its Articles (Hickman 2021).

In this paper, I offer an analytical and normative framework to re-visit the question of whether state parties should derogate or not. Methodologically, I argue that one should address substantive and empirical variables and concentrate on the disproportionate effects of the pandemic on minority and vulnerable groups (e.g. children and young people, asylum seekers, people with disabilities, women). With a view to addressing these effects, I then argue that proportionality assessment should be the basis for determining whether state parties ought to derogate or not. This is not only because proportionality’s very purpose is in part to track the effects of state interferences on minorities and vulnerable groups by measuring the disproportionate burden imposed on them, which speaks to the egalitarian foundations of human rights (Möller 2021), as I shall explain. It is also because proportionality assessment has very different requirements between limitation clauses built into the relevant Convention articles (e.g. Article 5, Articles 8–11) and the derogation clause (Article 15) under the ECHR. Surprisingly, while the emerging literature almost always mentions proportionality as an important component of the analysis, it does not investigate the extent to which each regime (derogation or limitation) better performs it, and why.

On this basis, I argue that not derogating — and staying within the realm of the Convention via the restriction clauses built into it — is decidedly better as a matter of proportionality tracking. Unlike Article 15 that almost only understands proportionality as *necessity*, proportionality requires public authorities to examine the egalitarian implications of restricting measures on rights and to justify these measures to their subjects in normative terms. In particular, the ECHR comprises a restriction clause of ‘necessity in democratic society’ (explicit in Articles 8–11) that examines how much burden imposed by an interference on *some* individuals is justifiable vis-à-vis *others*. I draw here from the philosophical literature on the “right to justification” conceived as built into the proportionality test (Möller 2012, Möller 2021, Kumm 2010, Forst 2010). Unlike derogation, limitation clauses have a much higher and systematic requirement of normative justification triggered by proportionality analysis. In this sense, proportionality serves to foster the egalitarian foundations of human rights and this fostering is of prime importance in the context of the pandemic.

## Debating (Non)-Derogation: the Textual Approach and Its Limits

The ‘lockdown’ measures (sometimes also called ‘mass quarantine’) imposed by European governments to combat the COVID-19 pandemic — in particular, during the first wave as of March to May 2020 — has undoubtedly interfered with a number of rights and freedoms protected by the ECHR. In the UK, for example, such measures were enforced mainly through the Health Protection Regulations 2020 (‘the Regulations’), which were introduced under the powers delegated by the Public Health (Control of Disease) Act 1984 (‘the 1984 Act’). These measures had a drastic impact on a range of ECHR Articles. For example, the exercise of the freedom of thought and religion (Article 9) was severely limited as the measures prevent the right of worshipers to attend or gather in any places of worship other than for funerals; the general public was prevented from gathering all together, which could constitute a deprivation of the right to liberty (Article 5); the interference with the right to association and assembly (Article 11) is similarly concerned; Article 2 of Protocol 1 (the right to education) was interfered with if children were denied basic education as a result of school closures; people suffering from physical and/or mental illness were heavily impacted, which raises the risk of an Article 14 violation (right to non-discrimination).

In order to justify these extraordinarily restricting measures, states have either triggered the derogation clause of the ECHR (Article 15), which allows them to temporarily suspend the application of the relevant Article(s) or has determined that these measures were justifiable based on the text of the relevant Article. In this first section of the article, I reconstruct the terms of the emerging (non-)derogation debate in the UK with respect to this dilemma. I emphasize the methodology adopted by the two main sides to the debate — and as such hope to raise an issue that is not distinctive of the British legal context but concerns broader issues of methodology and theory in (European) human rights law.

It will appear that the predominant approach to the (non-)derogation question is *textual*. Briefly, this approach understands the principled role of human rights adjudication as primarily concerned with determining the content and scope of a provision based on its semantic content — and this exercise sets out the perimeter for deciding the question (non-)derogation. This approach then helps decide whether the lockdown measures taken by state parties fall within one or the other provision. Central to the textual approach, therefore, is the assumption that the content of human rights can be set independently of the justification offered by the respondent state party for the interference. The seeds of this approach are not only found in judicial methodology, as we shall see. They also echo a philosophical conception of human rights understood as a subset of moral rights assigned to a limited domain of moral life. In this sense, this article also offers an occasion to test this overarching account cutting across human rights qua legal rights and human rights qua moral rights.

### Article 5

The debate on the question of (non-)derogation starts with a discussion of the textual basis of Article 5 ECHR (‘the right to liberty and security of the person’) and Article 15 ECHR (the derogation clause) under the ECHR. The key assumption here is that if the lockdown measures fall outside of the core content and scope of Article 5, triggering the derogation clause becomes the only available framework for complying with the ECHR. It is indeed generally assumed that Article 15 considerably enlarges the scope of what measures state parties can take (including an extensive margin of appreciation). Conversely, if the lockdown measures cannot meet the threshold of Article 15, it follows that these facts should be covered by Article 5. In the latter case, instead of *derogating* from rights state parties would only *limit* them and hence would not *violate* them.

In this textual context, the first specific issue in the debate pertains to the meaning of the terms in Article 5(e). In particular, this provision contains one limitation close that seems very relevant to the pandemic context, namely that “no one shall be deprived of liberty his save (…)”.

‘(e) the lawful detention of persons for the prevention of the spreading of infectious diseases, of persons of unsound mind, alcoholics or drug addicts, or vagrants’.

The key interpretive question under the clause is whether it applies only to people infected by the disease, or whether it could extend to the healthy subjects too. On this point, Greene for example contends that Article 5.1 (e) should only apply to ‘infected persons or persons who may be infected with necessary safeguards regarding the burden of proof required to fall under this category’ (Greene 2020, p. 267). It is important to note that Greene makes this point in the absence of any relevant case of the ECtHR on the matter — hence the speculative and hypothetical tone of the proposition. Indeed, his opponent in the debate Hickman recalls the case of *Austin v. United Kingdom* suggesting that Article 5 ‘will, in effect, be given a purposive construction so that it does not apply to all confinements where the type and nature of confinement differs from that intended to be addressed by Article 5’ (Hickman 2021, p. 7). In fact, Hickman argues, ‘there is nothing in the wording of art.5(1)(e) that limits its application to infectious persons and each of the points that we made in our blog provides a strong reason for reading the subparagraph in its natural and ordinary way’ (Hickman 2021, p. 8).

The case law of the ECtHR rapidly appears limited to gauge the correct content and scope of Article 5 in the context of the pandemic based on text. The case of *Einhorn v. Sweden* for example suggests that only infected individuals may be detained, whereas most of the most drastic COVID-19 measures applied to significantly large segments of state populations. Greene also emphasizes the distinction between lockdowns and quarantines in this respect: ‘lockdowns such as those seen during the COVID-19 pandemic in Europe, however, are different to quarantines in that they as a whole do not attempt to distinguish healthy persons from infected or possibly infected individuals’ (Greene 2021, p. 3). Logically, if lockdown measures go further and apply to everyone, that is something that would fall outside of the scope of Article 5, which then would justify triggering Article 15. Greene however acknowledges that ‘if the power were prescribed as the public authorities in question requiring “reasonable cause to believe” that individuals in a specific area should be deprived of their liberty, then it is quite possible that this construction may be compatible with Article 5.1(e)’ (Greene 2021, p. 3).

What slowly emerges from the discussion is that textually delineating the content and scope of Article 5 is a particularly arduous task. This difficulty could be bypassed by having a clear decision of the ECtHR. Yet, in the absence of an established Strasbourg jurisprudence on lockdown measures, Greene and Hickman mostly build upon resembling past cases of Article 5 and Article 15, the majority of which having only little resemblance to the COVID-19 pandemic (*Einhorn v Sweden*, for example, concerned the detention of a HIV-positive individual subject to compulsory isolation under Article 5). The only indicative evidence so far coming from Strasbourg is the case of *Terheş v Romania* (July 2021) where the Court found that the Romanian lockdown measures did not meet the threshold of deprivation of liberty under Article 5 and the application was deemed inadmissible (under Article 35 ECHR).

The absence of an established Strasbourg jurisprudence points to the limits of the textual approach — yet, these limits can be identified by other means. In particular, the use of counterfactuals for remedying the absence of judgements is quite illustrative — and for a textual approach quite unsatisfactory. In the words of Greene, *it ultimately depends* — ‘it depends on how the power is constructed and how it is used. This may not be a satisfactory answer to some, but detail and context matters with regards to understanding the nature and scope of human rights and measures that may potentially violate them. For this reason, thought experiments and hypothetical scenarios may not be the best method’ (Greene 2021, p. 3). Whether it extends to counterfactuals or remains within the limits of existing case law does not change much: implicit here is the idea that human rights norms (ought to) have a clearly delineated perimeter set by semantic content under the guidance of the authoritative adjudicator (the ECtHR and/or the UK Supreme Court in the case in the British context). I further elaborate on the limits of textualism in the next section.

### Article 15

The intricacies of Article 5 and the limited result of the textual approach are also salient in the second limb of the debate, namely the question whether Article 15 may be triggered in light of the scale of the pandemic. To recall, Article 15(1) ECHR stipulates that:

in time of war or other public emergency threatening the life of the nation any High Contracting Party may take measures derogating from its obligations under [the] Convention to the extent strictly required by the exigencies of the situation, provided that such measures are not inconsistent with its other obligations under international law.

The key textual question here is whether the pandemic facts meet the criteria of a ‘public emergency threatening the life of the nation’, which usually breaks down into the issues of the *magnitude* of the threat on the one hand and the *imminence* of that same threat on the other hand. Here again, Greene and Hickman look for guidance in the past cases where Article 15 was triggered, namely terrorist threats. On this point, Greene argues that the pandemic provides for an ‘ideal state of emergency’ — meaning that the pandemic measures went much further than any known case where Article 15 was applied. He argues that ‘the threat posed by a pandemic similar to COVID-19 therefore is several orders of magnitude greater than that posed by terrorism. To permit derogations in order to prevent an imminent eruption of an epidemic is not to water down the meaning of a “public emergency threatening the life of the nation”’ (Greene 2021, p. 6). Furthermore, unlike terrorist threats, the link between state intervention and the containment of the threat is a lot more direct: ‘our experience of exponential growth of infections from the COVID-19 pandemic serves to underlie the importance of such early intervention’ (Greene 2021:6). Greene articulates these arguments as an objection to Hickman’s argument that triggering derogation risk ‘watering down’ Article 15. Hickman builds an example where only a locality is concerned by lockdown measures, where the public health crisis is acute but where the ‘national emergency threatening the life of the nation’ as required by the text of Article 15 is clearly lacking. Under this scenario, Greene’s narrow interpretation of Article 5 that only infected people were confined would constitute a major risk for public health.

It transpires from this debate that the resources employed are emphatically textual: the analysis proceeds on the assumption that the meaning of human rights provisions is primarily to be grasped by one single resource, namely what is encoded in the text of these provisions. Now, it is widely accepted that textualism does not require adopting the literal meaning of a text as the line between semantics and pragmatics is at best not clearly drawn. Greenberg for instance defines textualism as ‘what it would be reasonable to take the speaker to communicate’ (Greenberg 2021), which makes textualism enmeshed with the intentions of the legislator (in this case, the Convention drafters). The end result would be ‘what a reasonable person, given the context, would take the legislature to have intended’ (Ibid.). Now, while it appears that the theoretical foundations of textualism are heavily disputed — for the purpose of this article it suffices to point to the absence of non-textual resources to address the question of (non-)derogation and to wonder why that is the case. I believe that this textual approach is at best too narrow to exhaust the conceptual, normative and empirical space that the question of (non-)derogation occupies in the context of the pandemic. The reasons for critically appraising the textual approach are both *internal* and *external* to the debate surveyed above. The internal issue with the textual approach is that it simply fails to deliver on its promise. As shown above, the scope of Article 5 and the manner in which the Court would approach it, in particular, are decidedly unclear. As Greene explains, we are situated in the ‘penumbra’ of rights:

No lawyer can definitively advise a government the unprecedented lockdown measures they introduced are compatible with art. 5.1(e) because, quite simply, until we get a definitive ruling on the scope of art.5.1(e) from the European Court of Human Rights, we cannot know. We are thus in the penumbra of possible meanings as to the scope of art. 5.1(e) (Greene 2021, p. 8).

One may very well reply that waiting for and ultimately obtaining a relevant ECtHR’s judgement will appease the concern. But the fact that the Court issues a judgement does not suffice to alleviate the limitations the textual approach. The broader and external issue is that, as indicated above, the exclusive concentration on the Convention’s text *ipso facto* filters which norms and facts are ultimately relevant to the legal analysis. While the debate heavily engages with factual features of the pandemic (as well as counterfactuals), ultimately the kind of facts discussed are canvassed by the explicit text of Article 5 and Article 15 (e.g. the existence of an ‘emergency’). But whether these are the only facts that matter to (European) human rights law — in the context of the pandemic specifically — is not a question that the textual approach can ultimately address precisely because it is tied to a limited textual perimeter. That is where, in my view, the textual approach suffers from a severe methodological and theoretical limitation — one that I further elaborate on below.

## A Fresh Start: Proportionality

Indeed, one way to further expose the limits of the textual approach to (non-)derogation is to show that it simply leaves out a vast portion of the practice of courts (with respect to the pandemic specifically, but with regard to human rights generally): even if we were to have a decidedly clear textual account of the rights’ content scope, courts would still have to determine whether, under the circumstances of the case at hand — the pandemic ones — state authorities may have sufficient reasons to limit or to restrict them (or, in the case of Article 15, to trigger derogation). In the case of Article 5, for example, individuals could be deprived of their rights and the state parties could still claim that the interference pursued a legitimate aim, namely prevention of the spreading of infectious diseases in accordance with Article 5.1(e), as we saw above. But the question remains: even if the state party pursued a legitimate aim, was the interference necessary and proportionate? It is a matter of ordinary the practice for the ECtHR to first lay down the ‘general principles’ extracted from past cases in order to define and interpret the content and scope of a given Article (for a standard example, see e.g. *Eweida and Others v. United* Kingdom, paras. 83–85).

To that limited extent, the textual approach is employed as a first methodological step. Yet, this textual background will be inert when courts turn their eyes to the justification offered by the state party for interfering with rights (Gerards and Senden 2009). The textual content and scope of a given right are distinct from the possible and legitimate grounds for restricting them. Indeed, when the Court examines the legitimate aim of an interference, it is hypothesizing its purpose, which entails engaging with normative reasoning proper. The Court does not determine purpose by appealing to textual elements. As Letsas explains, sometimes ‘the Court takes the moralistic preferences of the majority as being synonymous with “public morals” and thus constituting a legitimate aim’ (Letsas 2009, p. 121).

That is where the horizon of the (non-)derogation question can open up. The upshot here is that if one accepts that the textual approach is maladapted to fully address the pandemic, one can start including a much wider set of variables into the analysis, whether these are empirical or normative. This however presupposes abandoning the idea that human rights qua legal and moral rights necessarily have a distinctive semantic or normative content to them that can be grasped prior to placing these rights in the circumstances of their alleged violations. Rather, scholars such as Kumm and Möller have argued that the key question of human rights law and theory should focus on the conditions under which it is possible to restrict (or to derogate from) these rights. They precisely question the assumption that there a distinctive concept of human rights (legal or moral) that should occupy the central and often primary stage of adjudication. In the words of Kumm:

the problem is that actual human rights practice generally does not fit very well any limited domain conception of rights. Human rights claims do not, in legal practice, occupy a narrow domain limited to things fundamental, however that threshold might be understood. In legal and political practice rights claims occupy a domain that includes what might appear to be mundane and even trivial things (Kumm 2010, p. 134).

I believe that what Kumm and Möller say about human rights theory equally applies to the textual approach, namely the assumption that interpreting human rights provisions should start and remain focused on a discussion of their semantic content. Like Kumm and Möller, I believe that the question of (non-derogation) should be informed by the conditions under which individuals can have their rights interfered with. That is where proportionality enters the stage. The function of proportionality in human rights law is to determine the conditions for interfering with one or several protected rights. This implies that the proportionality test applies when courts or adjudicators have already established an interference with one or several rights based on established interpretive principles. They consider proportionality by turning to the normative grounds the respondent state had to interfere with these rights, the generic formulation of which is found in the text of the Convention. There are multiple formulations of the proportionality test depending on the constitutional or human rights system under consideration. It is generally taken to involve three distinct steps. Once an interference has been found prima facie, the court or adjudicator shall examine whether the policy or law interfering with one or several rights has a basis in law (‘prescribed by law’ in the text of the Convention). The second step is to assess whether the interference pursued a *legitimate aim*, which typically refers to a range of collective or public goods, such as national security and public health or morals. It is important to see that this step is *hypothetical*: courts are examining ‘whether there are any interests which are candidates for justifying the interference in the sense that it is not entirely implausible that they will at least be rationally connected to the policy’ (Möller 2012, p. 181).

These two criteria of proportionality are largely independent from the pre-existing (semantic or normative) content and scope of rights — they rather require placing these rights within the circumstances of their alleged violation and considering the reasons for interfering with them, which leads the Court to engage with normative reasoning. These criteria apply to both derogable and non-derogable rights of the Convention. It is often assumed that only derogable rights (Article 8–11 of the Convention) can be subject to proportionality analysis based on in-built restriction clauses — yet, the Court applies proportionality criteria to Article 5 too, which conventionally belong to the category of non-derogable rights. Yet, the Court has stressed in a seminal case that ‘regard must be had to a whole range of criteria such as the type, duration, effects and manner of implementation of the measure in question’ (*Engel v Netherlands*, para. 50). If that is correct, then in both Article 5 and 15, facts do not matter just to grasp the rights’ content and scope — they matter to evaluating if, for instance, state parties could have used a less invasive measure to pursue a legitimate aim justifying the interference. As such, proportionality broadens the analytical and evaluative horizon for deciding the question of (non-)derogation.

Now, one may readily object that most scholars debating the (non-)derogation question are acutely aware that proportionality constitutes an important part of the question to be addressed. Most authors — including Greene and Hickman — emphasize proportionality throughout their contributions to the debate. In fact, Article 15 places crucial proportionality constraints on what state parties may do when they trigger the derogation clause — the criterion of ‘strictly required by the exigencies of the situation’ in particular suggests that proportionality applies across the board. Hickman for example writes that ‘this standard has been held to bring a proportionality analysis into play. This form of reasoning bears a clear resemblance to the way that the Court has given the scope of each of Arts 8–11 a broad interpretation and applied the concept of proportionality to test whether measures are “necessary in a democratic society”. This enables the concept of proportionality to be applied in a wide range of circumstances and affords that concept the central role in determining the legality of state action under the convention’ (Hickman 2021, p. 4).

One may therefore initially doubt that it makes any difference to our assessment of (non-)derogation if in fact the proportionality requirements are part of the analysis. Dzehtsiarou for instance writes that ‘proportionality analysis can accommodate the measures adopted to fight health emergencies and its mechanism can account for the special circumstances of the COVID-19 pandemic. Article 15 does not change much of the legal analysis here except broadening an already broad scope of the margin of appreciation’ (Dzehtsiarou 2021: 3). On this view, proportionality will only be marginally different between restriction (under Article 5) and derogation (under Article 15). Dzehtsiarou adds that ‘formal derogation will not change the scope of acceptable proportionality significantly (…). Again, some adjustment of the margin of appreciation is possible due to the declaration of emergency but this adjustment will be minimal’ (Dzehtsiarou 2020: 10). If proportionality applies across the board with little difference across provisions, then why should we explore it further?

I believe that things become a lot less straightforward when one engages with the very concept and purpose of proportionality. Indeed, the debate surveyed above spills very little ink on proportionality as a concept and pays little attention to the theoretical work on it (Kumm 2015, Kumm 2016, Möller 2012, Möller 2021) in recent years. This is highly surprising as the rise of proportionality has been understood as a paradigmatic shift in constitutional theory — and this has implications precisely for the use of a textual approach to adjudication. Before returning to the pandemic, therefore, it is worth pondering what — beyond the context of (non-)derogation — this paradigmatic shift implies as a matter of theory and methodology in (European) human rights law. In nutshell, the rise of proportionality has been defined as a turn from a ‘culture of authority’ to a ‘culture justification’ as defined by Cohen-Elyia and Porat (Cohen-Elyia and Porat 2017). In their words, ‘a culture of justification derives from the court’s role and from the idea that government action is not legitimate when it is not justified’ (Cohen-Elyia and Porat 2017, p. 134). It is not just the authority of the text that is concerned here. The Court may also exemplify a culture of authority by relying on an established method of adjudication (e.g. ‘evolutive and dynamic’, ‘autonomous concepts’, ‘practical and effective’ in the context of the Court). The evolutive and dynamic approach would exemplify a culture of authority by ultimately relying on an authorized source of international law (Article 31(3) of Vienna Convention on the Law of Treaties) (Forowicz 2010, p. 269–70) rather than offering a normative justification for the interference based on its varying effects on individuals.

On the view that normative justification matters to the egalitarian foundations of human rights, it is at least narrow to limit the question of (non-)derogation to the text of Article 5 and Article 15, respectively. Indeed, Möller and others suggest that the right to liberty in particular should be a general right without set-in-stone boundaries, thereby making proportionality the device that will operate the test of legality constructed as a test of justification: ‘the effect of this is that a great number of state acts interfere with a protected right and thus trigger the duty of justification and proportionality analysis’ (Möller 2021, p. 343). From this perspective, it remains surprising that most participants in the debate surveyed above do not seem to fully consider the turn from authority to justification in constitutional and human rights adjudication — and when they do, they do not seem to draw implications of the turn for how one principally approaches questions of judicial methodology (Greene 2020, p. 45). Proportionality is overall taken as a necessary component of legality under the ECHR but not necessarily one that marginalizes textual analysis — and as we have seen, that proportionality is operative under Article 5 and Article 15 too. Yet, proportionality is only marginally sensitive to the textual dimension of rights — the analysis rather proceeds on a substantive, normatively thick basis that is reinforced by the adversarial nature of the reasoning in the test performed by the ECtHR. If we take the three usual steps of the test as worded under the ECHR (‘prescribed by law?’, ‘legitimate aim?’, and ‘necessary in a democratic society?’), it will be difficult, for example, to textually confine the kind of legitimate aim(s) that a state party may pursue in interfering with rights. As a matter of practice, the ECtHR defines legitimate aims by appealing to normative reasoning, not textual. Furthermore, the necessity stage of analysis is another example where factual rather than textual elements come to the fore, as we shall see below.

More importantly for our purposes, the final stage of balancing in the proportionality test is one where text is unhelpful: courts cannot balance strictly defined words against each other but need to address the values and interest(s) underlying words — in other words, proportionality requires both the respondent state party and the Court to engage in an exercise of normative justification in light of the grounds for limiting or restricting them. As Webber explains (in the constitutional context), ‘because rights are constituted by their limitation, the process of practical reasoning by which those limitations are identified must appeal to reasons, which, if valid and when taken together, can justify the specification of the right’ (Webber 2009, p. 181). This is not just a theoretical argument: it is abundantly clear that the Court appeals to normative reasoning in its ordinary practice when it applies proportionality, as I exemplify below. The question rather is as follows: which normative values does the Court use, and which ones should it appeal to? I suggest viewing the last, balancing stage of proportionality as engaging with the broader and substantive value of moral equality. The core principle here is that interfering with the freedom of individuals (e.g. with Article 5 as defined above) for the sake of pursuing a legitimate aim should ensure that the burden of interfering is not asymmetrically imposed across the individuals and the groups subject to the interfering norm. I am here relying on the work of Möller, for example, who precisely defines ‘fundamental equality’ as one of the ‘dignitarian principles’ of human rights, legal and moral. In his view, proportionality requirements ‘ensure that a policy does not impose a burden on a person which treats their individual interests as less important than other people’s’ (Möller 2021, p. 363). This leads to the operative idea that courts should span the effects of government policies through the prism of equality, not only legality (‘prescribed by law’) and legitimacy (‘legitimate aim’). But how does a court exactly operate this egalitarian function? Equality is most visible when the ECtHR examines whether the interference was ‘necessary in a democratic society’, which is again the last, balancing stage of proportionality analysis (often called proportionality stricto sensu). Now, it appears that this egalitarian clause is most active in the Court’s practice under Articles 8–11 but often makes its way into other provisions as interpreted by the Court.

Is this egalitarian clause also operative under Article 15? This is where one may start noticing discrepancies in proportionality requirements across the Convention articles, which seem to go largely unnoticed in the (non-)derogation debate surveyed above. Indeed, it appears that under Article 15, the Court extensively focuses on the issue of necessity — in direct reference to the clause of ‘strictly required by the exigencies of the situation’ — and, as such, tends to avoid going into egalitarian assessment. In particular, while the Court will usually leave it to the state to determine the existence of a national emergency (typically, via the margin of appreciation), it will scrutinize whether the steps taken by the state were strictly necessary in relation to this self-established emergency. For example, in the notorious case of *Askoy v. Turkey*, in which a Turkish citizen was detained on suspicion of aiding and abetting PKK terrorists, the Court emphasized necessity throughout all its reasoning to its conclusion:

The Court has taken account of the unquestionably serious problem of terrorism in South-East Turkey and the difficulties faced by the State in taking effective measures against it. However, it is not persuaded that the exigencies of the situation necessitated the holding of the applicant on suspicion of involvement in terrorist offences for fourteen days or more in incommunicado detention without access to a judge or other judicial officer (ECtHR, *Aksoy v. Turkey*, App. No. 21987/93, 18 December 1996, para 78).

Similarly, in *Mehmet Hasan Altan v. Turkey*, also a case of pre-trial detention without reasonable suspicion, the Court’s reasoning is confined to necessity considerations — again, informed by the reference to ‘exigencies of the situation’:

Having regard to Article 15 of the Convention and the derogation by Turkey, the Court considers, as the Constitutional Court did in its judgement, that a measure of pre-trial detention that is not “lawful” and has not been effected “in accordance with a procedure prescribed by law” on account of the lack of reasonable suspicion cannot be said to have been strictly required by the exigencies of the situation (ECtHR, *Mehmet Hasan Altan v. Turkey*, App. No. 13237/17, 10 September 2018, para 140).

This thin account of proportionality qua necessity should be distinguished from a thick account that includes the egalitarian clause. There is a conceptual distinction between the two: to assert that a state measure — e.g. a decision of pre-trial detention — is necessary to achieve the aim in question is logically distinct from examining the potentially disproportionate burden of that same measure across a given population. Whether a pre-trial detention of X (via a law, policy or decision of state Y) is necessary to prevent terrorist action Z engages with (hypothetical) causation and remains logically independent from the question of how much burden the law, policy or decision of state Y imposes on its population. Indeed, the latter is *other-regarding* as the analysis requires incorporating egalitarian, and therefore moral, variables into the analysis. Not doing so disregards the moral premise that individuals are equal and therefore that if they are to be treated differently, a sufficiently strong case needs to be made.

The case of *A v. UK* (2009) addressing the pre-trial detention of suspected terrorists is quite illustrative here. This is allegedly the only case where the Court incorporated equality as a proportionality variable under Article 5. Indeed, the Court emphasized both issues — necessity and equality — and finds a violation on the ground that the measure in question is disproportionate on egalitarian terms. The issue of necessity is addressed first by examining the nature of the threat and the seemingly bizarre claim of the UK government that this threat can only derives from foreign nationals — and in doing so, the UK House of Lords is examining factual and causal features of the social world:

While the threat to the security of the United Kingdom derived predominantly and most immediately from foreign nationals, some of whom could not be deported because they would face torture or inhuman or degrading treatment or punishment in their home countries and who could not be deported to any third country willing to receive them, the threat to the United Kingdom did not derive solely from such foreign nationals (ECtHR, *A v. United Kingdom*, App. Nos 100/1997/884/1096, 23 September 1998, para. 31).

Yet again, this empirical and causal issue remains independent from the question of what one should do once the threat is empirically defined. The egalitarian assessment starts when one considers why only foreign nationals should carry all the burden of the fight against terrorism as only they will be subject to these harsher measures. In order to support the latter statement, one must insert a measure of equality that precisely and comparatively examines the burden across individuals and groups. In this specific case, it could very well be that the threat mostly derives from foreign nationals, but it remains unjustifiable — from the standpoint of equality — to apply this right-interfering norm to them as if only they had to carry all the burden. I take the Court to say precisely that when it held that:

In conclusion, therefore, the Court, like the House of Lords, and contrary to the Government’s contention, finds that the derogating measures were disproportionate in that they discriminated unjustifiably between nationals and non-nationals. It follows that there has been a violation of Article 5 § 1 in respect of the first, third, fifth, sixth, seventh, eighth, ninth, tenth and eleventh applicants. (*Ibid., paras 189–190).*

This case exemplifies that thick proportionality is not necessarily absent from the Court’s review of the derogating measures under Article 15, but it is doubtful that the concentration on the ‘exigencies of the situation’ is likely to trigger it. Moreover, it seems that the Court relies on (and agreed with) the UK House of Lords’ egalitarian argument rather than derived it from the text of the Convention. It remains that in most Article 15, proportionality qua necessity has prevailed. It is also clear that the Court will be less likely to extend its scrutiny over thicker (and egalitarian) proportionality if it accords a margin of appreciation as to the existence of an emergency.

It worth summing up the claims made so far in this article before returning to the pandemic. First, the textual approach is not only maladapted to legally appraise the pandemic in the absence of clear guidance from the Court — and even if there were any clear guidance, it does not follow that the textual approach would per se suffice. Indeed, such an approach also marginalizes the key issue of proportionality by assuming that proportionality operates similarly across the relevant articles, as our review of the literature has revealed. Second, I have argued that proportionality testing has both thin (necessity) and thick (equality) components that are logically distinct. The latter includes an egalitarian analysis that examines if the burden for pursuing the legitimate aim — in the cases surveyed above, the aim of fighting terrorism and preventing attacks — is equitably distributed across individuals and groups. This normative assessment speaks to the broader function of justification that the rise of proportionality signals. More generally, I emphasized that proportionality is methodologically sensitive to much wider set of variables than the textual approach because it requires the Court to constantly assess comparisons across different measures (necessity) and subjects (equality). I shall return to the context of the pandemic via this last point in the next section.

## The Covid-19 Pandemic and the ‘Disproportionate Burden’

It has become increasingly documented that the radical measures taken by state parties to combat COVID-19 pandemic — in particular, the lockdown measures via social distancing rules — have significantly exacerbated existing inequalities across Western Europe. Minority and vulnerable groups (e.g. children young people, women, prisoners, asylum-seekers, people with disabilities) have been affected in multifaceted ways by these measures. Indeed, the effects point to the worsening of existing structural inequalities that date back to far beyond the pandemic. The point of this section is not to empirically investigate these effects or even to review the extensive empirical literature that is emerging across a wider range of disciplines. Rather, I aim to show how proportionality can effectively the egalitarian effects of rights’ interferences, which can help us determine which of the two regimes (restriction or derogation) is best suited. Now, just to recall, the textual approach is methodologically limited in the facts it can examine — the text filters them. My approach based on proportionality allows to look at a much wider factual, conceptual and normative horizon. As an example, the drastic lockdown measures introduced during the two first waves of the pandemic had a vastly disproportionate effect on two minority groups, namely (i) people with disabilities and (ii) children and young people. Recent public health and education research shows that social distancing measures, for instance, have impacted the delivery of care (to people with disabilities) or education (to children and young people) in significant ways and at scale.

In the case of people with disabilities, for example, research shows that social distancing measures have made at home care, therapy and learning extremely difficult. Shakespeare et al. explain that for instance, ‘routine physiotherapy, speech and language therapy and occupational therapy were cancelled, causing particular problems for young disabled people. Attempts to replicate these therapies either via video conference or phone were not perceived to be particularly successful’ (Shakespeare et al. 2021, p. 5). Research also shows that people with disabilities turned to informal caregivers and support — in particular, charities — as a substitute for public services. Again, it is essential to grasp that these effects build upon prior, structural inequalities and discriminations across the UK. A wide survey led by the city of Manchester found that 83% of the respondents are generally worried about how they would be treated in hospital because of general attitudes to disability. A total of 76% of the respondents are generally dissatisfied with the help provided by the government (Greater Manchester Disabled Peoples’ Panel 2021).

The effects of lockdown measures on children young people (age 12–24)’s health and development are no less worrying — in particular, when these effects also apply to learning processes and outcomes. The vast school closures experienced across Europe implied that the usual type of social and learning environment involving one-to-one interactions with mentors and peer groups suddenly and abruptly stopped. The effects on the mental health of pre-adolescents have proven to be the most salient. Recent research shows a severe deterioration in mental health among pre-adolescent children in the UK, with for example a 20% increase in hyperactivity/inattention and a 35% increase in conduct problems (Waite et al. 2021). The mental health of young people deteriorated more than any other group during the first lockdown according to a recent study conducted by the British academy. The study also points to the nexus between the impact on educational outcomes and other parameters such as domestic violence: ‘gaps in educational achievement for children and young people, exacerbated by school closures and varied access to online education tools, have been shown to heighten risk of abuse and neglect as well as exposure to domestic violence’ (British Academy 2021, p. 34).

This short overview indicates that the effects of lockdowns on minority groups such as people with disabilities as well as children and young people are both direct and indirect. They not only pertain to an immediate and drastic impact on people’s ordinary life and well-being. These effects may well continue affecting individuals in the longer term, in particular when it comes to education or therapy outcomes. Now, the purpose of mapping these effects is then is to examine if and how (European) human rights law can and should pay attention to them. As argued above, the extent to which the Court can be sensitive to the facts will depend on whether the purpose of human rights review is informed by a framework of authority of justification. A framework of justification would be only marginally sensitive to the text of the relevant Convention articles, as we have seen. Rather, the process of adjudication would concentrate on the conditions under which rights may be restricted — and therefore *whose* rights would be restricted as a result of the interference(s). As we saw above, proportionality is a formal device that requires state party to offer this justification — namely, that it does not ‘impose a burden on a person which treats their individual interests as less important than.

other people’s’ (Möller 2021, p. 363). The text of the right to freedom (Article 5) or the right to education (Article 2 Protocol 1) is only the starting point of the analysis. Proportionality requires that one looks at the effects of the lockdown measures and comparatively so. The right to non-discrimination (Article 14) can further support that approach.

I suggest that there is significant room within the Convention to address and mitigate these effects through proportionality analysis — provided that we do pay utmost attention to what proportionality exactly does and why. To recall, under the approach of proportionality qua equality the question for the Court will not only be whether the lockdown measures effectively and uniquely achieve its aim of containing the virus and preserved the operative capacities of the health system (proportionality qua necessity). It will also consist of scrutinizing if and how all individuals and groups share the burden of pursuing that legitimate aim equitably. This is usually addressed by the Court in the last part of the proportionality test — often called proportionality stricto sensu. Having demonstrated that the right-interfering norm was ‘prescribed by law’ and pursued a ‘legitimate aim’, state parties have yet to demonstrate whether the right-interfering norm imposed was ‘necessary in a democratic society’ and whether it generated a disproportionate burden upon the applicant. As such, proportionality qua equality is principally equipped to trigger an analysis of the effects of the pandemic surveyed above. If this is correct, then the question of (non-) derogation ought to be informed by these both factual and egalitarian considerations, not only textual ones.

## Conclusion

The article was an attempt to clarify the issue of the legality of lockdown measures and, in particular, the dilemma of (non-)derogation. Specifically, it aimed to broaden the horizon of analysis by extending the analytical lens to proportionality. I started by showing that the debate — predominant in the UK — is limited by a textual approach, which suffers from both internal and external limitations. The internal limitation is that a clear guidance from the Court is lacking. The external limitation amounts to dismissing an increasingly salient and important component of human rights adjudication, namely proportionality — a component that needs to be explored conceptually before it can shed some light on the (non-)derogation question. This article hence turned to the paradigmatic shift between a culture of authority and a culture of justification — and, as far as the latter is concerned, to the egalitarian function of proportionality. In the Convention itself, this function is performed by the last, balancing step of ‘necessity in a democratic society’. On that basis, I argued that remaining within the ECHR — and not derogation — is decidedly better because it ensures a much higher degree of justification than the derogation clause. While textualism limits justification to the semantic content of the provision, proportionality ensures a further and substantive layer of justification as to why a right’s limitation is justifiable to its subjects, defined as free and equal. It asks us to examine the disproportionate burden that some, rather than other, will have to carry as a result of interferences with rights. That is a normative justification. My argument also calls for further analysis of case law: did non-derogation states apply proportionality accordingly? In December 2020, the Court of Appeal in England reviewed the lockdown legislation and established that:

there can be no doubt that the regulations did constitute an interference with article 8 but it is clear that such interference was justified under article 8(2). It was clearly in accordance with law. It pursued a legitimate aim: the protection of health. The interference was unarguably proportionate (*R (Dolan) v Secretary of State for Health and Social Care (Dolan)* [2021] 1 All ER 780).

This finding further illustrates the current confusion pertaining to the legality of lockdown measures adopted by European states to combat the pandemic. We saw that the Court in *A v. UK* did appeal to egalitarian consideration under Article 5. In *Dolan*, the UK did not derogate from the ECHR and therefore aimed to justify the restriction by proportionality analysis — but what proportionality? The passage above clearly illustrates the thin model of proportionality qua necessity and legitimacy, one that glosses over the key question of proportionality qua equality. It is therefore urgent that scholars further press judiciaries to clarify the proportionality stakes attached to the (non-)derogation question — and when they opt for non-derogation, that judiciaries apply proportionality analysis comprehensively and consistently. This article aimed at offering some support to this scholarly enterprise.

## Footnotes

1 https://www.coe.int/en/web/conventions/derogations-covid-19

## References

[CR1] Cohen-Eliya M (2003). Proportionality and constitutional culture.

[CR2] Dzehtsiarou K (2020). Article 15 derogations: are they really necessary during the COVID-19 pandemic?. Eur Hum Rights Law Rev.

[CR3] Forowicz M (2010). The reception of international law in the European Court of Human Rights.

[CR4] Forst R (2010). The justification of human rights and the basic right to justification: a reflexive approach. Ethics.

[CR5] Gerards J, Senden H (2009). The structure of fundamental rights and the European Court of Human Rights. Int J Cons Law.

[CR6] Greenberg M (2021) Legal interpretation. In Edward N. Zalta (ed), The Stanford Encyclopedia of Philosophy, Fall 2021 edn. Metaphysics Research Lab, Stanford University, 2021. https://plato.stanford.edu/archives/fall2021/entries/legal-interpretation/

[CR7] Greene A (2020). Emergency powers in a time of pandemic.

[CR8] Greene A (2021). (2021) Derogations, deprivation of liberty and the containment stage of pandemic responses. Eur Hum Rights Law Rev.

[CR9] Hickman T (2020). The coronavirus pandemic and derogation from the European Convention on Human Rights. Eur Hum Rights Law Rev.

[CR10] Kumm M (2010). The idea of socratic contestation and the right to justification: the point of rights-based proportionality review. Law Ethics Hum Rights.

[CR11] Letsas G (2009). A theory of interpretation of the European Convention on Human Rights.

[CR12] Manchester Community Central (2020) Report: GM Big Disability Survey COVID-19 Report. https://manchestercommunitycentral.org/news/report-gm-big-disability-survey-covid-19-report. Accessed 7 June 2022

[CR13] Möller K (2015). The global model of constitutional rights.

[CR14] Möller K (2021). Beyond reasonableness: the dignitarian structure of human and constitutional rights. Can J Law Jurisprud.

[CR15] Shakespeare T (2021). Disabled people in britain and the impact of the COVID-19 pandemic. Soc Policy Adm.

[CR16] The British Academy (2021) The COVID decade: understanding the long-term societal impacts of COVID-19. https://www.thebritishacademy.ac.uk/publications/covid-decade-understanding-the-long-term-societal-impacts-of-covid-19/. Accessed 7 June 2022 https://www.coe.int/en/web/conventions/derogations-covid-19

[CR17] Waite P et al. (2021) How did the mental health symptoms of children and adolescents change over early lockdown during the COVID-19 pandemic in the UK? JCPP Advances 1(1). 10.1111/jcv2.12009 10.1111/jcv2.12009 PMC8206715 34485988

[CR18] Webber G (2009). The negotiable constitution: on the limitation of rights.

